# Responding to the environmental emergency through education: the imperative for teacher support across all subjects

**DOI:** 10.14324/111.444/ucloe.1987

**Published:** 2024-07-24

**Authors:** Kate Greer, Nicola Walshe, Alison Kitson, Justin Dillon

**Affiliations:** 1 IOE, UCL’s Faculty of Education and Society, University College London, London, UK

**Keywords:** education, climate change, the environment, knowledge, teachers, professional development, climate

## Abstract

The most recent Intergovernmental Panel on Climate Change report sets out sobering scenarios about the future for our young people and appeals for ‘deep, rapid, and sustained reductions in greenhouse gas emissions’. Although technological responses are essential to achieve these reductions, technocratic solutions alone will not solve the environmental emergency; a widespread societal transformation is needed. Education can play a profound role in this transformation as it is fundamental to building a society with knowledge, skills and motivation to tackle climate change so as to regenerate ecological and social systems. This commentary reflects on multiple dimensions of education’s role, focusing particularly on schools and the important contribution that all subjects can make towards developing interdisciplinary, complex understandings of the environmental emergency and living more sustainably. Drawing from a recent nationwide survey of teachers in England carried out by the UCL Centre for Climate Change and Sustainability Education, we highlight a troubling lack of engagement in formal professional development related to climate change and sustainability, even amongst a ‘climate change engaged’ cohort of teachers, and the imperative for comprehensive professional learning for teachers from across all subjects and year levels.

The latest Intergovernmental Panel on Climate Change report [1] set out sobering scenarios about the future for our young people, predicting that ‘adverse impacts from human-caused climate change will continue to intensify’ (p. 6). In 2024, UN Secretary General, António Guterres sounded the alarm about greenhouse gas emissions continuing to rise, not fall as is required if humanity is to meet the Paris Agreement target and limit long-term global heating to 1.5 °C [2]. Yet, assessments of the UK’s climate change response are gloomy. The independent Climate Change Committee has highlighted a ‘lack of urgency’ ([3], p. 14) in the UK Conservative Government’s response and an ‘overly narrow approach to solutions which, crucially, does not embrace the need to reduce demand for high-carbon activities’ ([3], p. 13). The mandate for drastic greenhouse gas emission reductions is unequivocal [1] and technological responses are essential. However, a narrow view that focuses on technocratic solutions will not solve the environmental crisis; instead, a widespread transformation which recognises the interdependence of climatic, ecological and social systems is needed [1]. All sectors of society have a role to play in learning how to avoid reproducing the damaging social structures and attitudes that have led us into the crisis, to develop the capabilities to repair the environmental damage caused and to forge sustainable ways of living.

Education, which can be understood broadly and enacted in formal and informal settings, has a role to play in this transformation. Education institutions, including schools, can play a part in greenhouse gas emission reductions that are so urgently needed through energy, transport, procurement and food policies and actions; indeed, according to the Department for Education, ‘schools and universities represent 36% of total UK public sector building emissions’ [4]. Schools can promote more sustainable lifestyles by reducing resource use, reusing and recycling, growing food and encouraging plant-based diets. They can contribute to biodiversity regeneration by conserving and rehabilitating pockets of estate grounds or local landscapes. Such actions, which take leadership, commitment and investment, can position schools as demonstration and learning hubs for students and staff, parents and school communities.

Alongside such practical actions, is the contribution that schools can make towards building a society with the knowledge, skills and motivation to boldly tackle the environmental emergency and respond to the threat of *un*-sustainability [5]. In broad terms, this first requires recognition of the crisis, followed by both subtle and substantial reorientations across the education system, towards ecologically (rather than economically) centred policies and the promotion of multi-species justice. It requires a shift away from producing and reproducing forms of knowledge and culture that have led to the crisis and are in service of economies that are built on extractive processes, towards diverse types of knowledge [1] and culture that is in service of all species on Earth [6]. This argument will be familiar to environmental education scholars who have long advocated for interweaving socio-emotional and indigenous knowledge with the disciplinary knowledge and skills that tend to be taught in schools.

Classroom teaching is, arguably, the principal site for enacting climate change and sustainability education in formal, school-based settings. Researchers have identified the ongoing prevalence of subject-specific knowledge-led approaches, especially science [7, 8]. In England, where schools are dominated by curriculum, exams and inspection [9], the few direct mentions of climate change in the National Curriculum are concentrated within geography and science [10, 11]. Whilst advocating for broad, expansive educational approaches to the environmental emergency and transformation across the education system, UCL’s Centre for Climate Change and Sustainability Education (CCCSE) recognises this as the current context in which schools operate. Therefore, given that immediate responses to the crisis are needed, our principal aim is to support teachers to incorporate climate change and sustainability into their subject-based teaching practices.

The work of CCCSE, which was established in 2022, has been informed by an initial study [12] which identified inconsistencies in the quality and availability of climate change and sustainability education in England, and noted that teachers sought more ‘time, confidence and resources’ (p. 17) to incorporate climate change into their teaching, a finding that is supported elsewhere [13–15]. However, little is known about the types of professional learning that teachers have participated in, the types of support they seek, and for whom this support would be most helpful. That is why CCCSE set out to investigate teachers’ perspectives of climate change and sustainability education, with a particular focus on their teaching practice and professional learning, through a nationwide survey (supported by the UCL IOE Strategic Investment Fund).

The results from our initial analysis [16] can be viewed as representing a ‘climate change engaged’ cohort of teachers. We invited responses from teachers of all subjects and phases of education; of the 870 respondents, 81% reported that they ‘sometimes’, ‘often’ or ‘very often’ incorporate climate change and sustainability in their teaching. Whilst the cohort included responses from teachers who taught subjects from across the National Curriculum, climate change and sustainability were reported as most commonly being incorporated into geography and science in secondary settings. This finding is unsurprising insofar as it correlates with how climate change is framed in the National Curriculum; yet it could also reflect teachers’ conceptions of climate change education whereby they are teaching relevant material, without badging it as such. Viewed another way, this could be concerning because climate change, and the broader environmental emergency of which it is a part, is too complex to narrow down to the disciplines of science and geography [17]; all disciplines contain knowledge and skills that can contribute towards developing the complex, interdisciplinary understandings of the environmental crisis and how we can live more sustainably [18]. A constructive response will require creativity, criticality, empathy and knowledge from across disciplines and epistemologies.

Our survey findings are most enlightening when it comes to professional learning which, following Pollard et al. [19], we understand as a broad and flexible concept to describe learning that occurs across the professional life of the teacher, and can include formal professional development activities or episodes (p. 506). We found that less than half of the teachers involved in the survey considered that they had participated in formal professional development related to climate change and sustainability. Of those who had, less than 13% considered that there had been a focus on climate change and sustainability in their Initial Teacher Education, whilst the most commonly reported type of professional learning was ‘self-taught’. The distinct lack of engagement in formal professional development that was reported by our ‘climate change engaged’ cohort of teachers is troubling and merits further exploration, as does the nature of the broader professional learning which teachers undertake.

Confident and capable teachers are crucial to helping young people to understand the Earth as an interconnected system of which they are a part, while developing the agency and capabilities to act for the environment and for a socially just society. That is why, in response to the survey findings, the CCCSE is developing a professional development programme to support teachers of all subjects and year levels to incorporate climate change and sustainability into their practice. The programme – *Teaching for Sustainable Futures* – comprises free online professional development modules for teachers that include films, activities and resources that teachers can incorporate into their teaching practice. Modules are currently available in history, geography, English and mathematics, with science and creative arts modules in development.

The modules bring disciplinary, everyday and indigenous knowledge together; for instance, in the secondary English module teachers are introduced to a spoken-word poem performed by Kathy Jetñil-Kijiner, of Marshallese ancestry, and Aka Niviâna, an Inuk woman. Teachers are asked to note their personal responses to the text before reflecting on its potential role for supporting the development of core English skills specified in the National Curriculum. The modules also aim to support teachers explore possible futures with their students; in the history modules, for example, a focus on industrialisation (secondary) and trees (primary) culminates in renewable energy technologies and woodland conservation, respectively.

While the provision of professional development which can support effective classroom teaching is important, CCCSE also recognises the ongoing impact that current assessment and inspection regimes have on teachers’ practice. That is why, alongside *Teaching for Sustainable Futures*, the CCCSE’s work programme extends to research into climate change and sustainability policy and practice, and national and international knowledge exchange and policy dialogue, with the aim of contributing to advancing effective climate change and sustainability education in England, and further afield.

## Data Availability

The datasets generated during and/or analysed during the current study are available from the corresponding author on reasonable request.
